# An unusual occurrence of multiple primary malignant neoplasms: a case report and narrative review

**DOI:** 10.3389/fonc.2024.1381532

**Published:** 2024-07-17

**Authors:** Rawand Qasim Salhab, Zeina Ihab Ghazaleh, Wadee Barbarawi, Riyad Salah-Aldin, Hani Hour, Raghad Sweity, Izzeddin A. Bakri

**Affiliations:** ^1^ Medical Research Club, Faculty of Medicine, Al-Quds University, Jerusalem, Palestine; ^2^ Department of Urology, Al-Makassed Islamic Charitable Hospital, Jerusalem, Palestine; ^3^ Department of Oncology, Beit Jala Hospital, Bethlehem, Palestine; ^4^ Department of Pathology, Al-Makassed Islamic Charitable Hospital, Jerusalem, Palestine

**Keywords:** multiple primary malignant neoplasms, multidisciplinary team, basosquamous carcinoma, prostatic adenocarcinoma, hepatocellular carcinoma, clear renal cell carcinoma

## Abstract

**Introduction:**

Multiple primary malignant neoplasms (MPMNs) are cancers presenting distinct pathological types that originate from different tissues or organs. They are categorized as either synchronous or metachronous. Nowadays, the incidence of MPMN is increasing.

**Patients and methods:**

We present a case of a 71-year-old male patient with a medical history of hepatitis B and a family history of breast and endometrial cancers. The patient reported a nasal tip skin lesion with recurrent bleeding, and the history disclosed lower urinary tract symptoms. Further investigations revealed the coexistence of four primary cancers: basosquamous carcinoma of the nasal lesion, prostatic adenocarcinoma, hepatocellular carcinoma, and clear cell renal cell carcinoma.

**Results:**

A multidisciplinary team cooperated to decide the proper diagnostic and therapeutic modules.

**Conclusion:**

To the best of our knowledge, the synchronization of these four primary cancers has never been reported in the literature. Even so, multiple primary malignant neoplasms, in general, are no longer a rare entity and need proper explanations, a precise representation of definition and incidence, further work-up approaches, and treatment guidelines as well.

## 1 Introduction

Multiple primary malignant neoplasms (MPMNs) are defined as two or more primary malignant tumors, each presenting a distinct pathological type that originates from a different tissue or organ (1, 2). MPMNs are categorized as either synchronous or metachronous by the Surveillance Epidemiology and End Results (SEER) Program, the International Association of Cancer Registries (IACR), and the International Agency for Research on Cancer (IARC) based on the period between each cancer diagnosis (3).

MPMNs were first described by Billroth in 1889 (4) and extensively studied by Warren and Gates in 1932 (1). Since then, MPMNs have been widely explored, and many cases have been reported. Despite it being considered rare, MPMN incidence is increasing due to the evolution of diagnostic methods and screening programs in addition to improved treatment modalities, resulting in enhanced survival rates for cancer patients (5, 6). Recent literature mentioned incidence of 2%–17%.

There are multiple theories discussing potential risk factors: family history, genetic defects, environmental factors, and previous primary cancers in the same patient, among others (7–9). Regarding the tumors’ origin, the theory is that they occur in a random manner (7). A comprehensive diagnostic approach is essential for primary tumor evaluation and early detection of possible consecutive neoplasms. Regarding treatment decisions, multidisciplinary team (MDT) is preferred for better evaluation of therapeutic choices and prioritization decisions.

In this article, we present a 71-year-old male patient with four primary malignancies: basosquamous carcinoma of the nasal tip, prostatic adenocarcinoma, hepatocellular carcinoma, and clear cell renal cell carcinoma. In addition, we provide a narrative review of the current state of knowledge in MPMNs. To the best of our knowledge, the synchronization of these four primary cancers has never been reported in the literature.

## 2 Case presentation

A 71-year-old married male patient presented to our hospital complaining of a nasal tip skin lesion for 18 months. His past clinical history is remarkable for hypertension, recurrent urinary retention, a 5-year history of hepatitis B that has been treated with lamivudine, and a surgical history of appendectomy, with no known drug or food allergies. His family history is significant for breast and endometrial cancer in the patient’s sister. The patient is not a smoker or an alcoholic and works as a driver.

**Figure 1:**
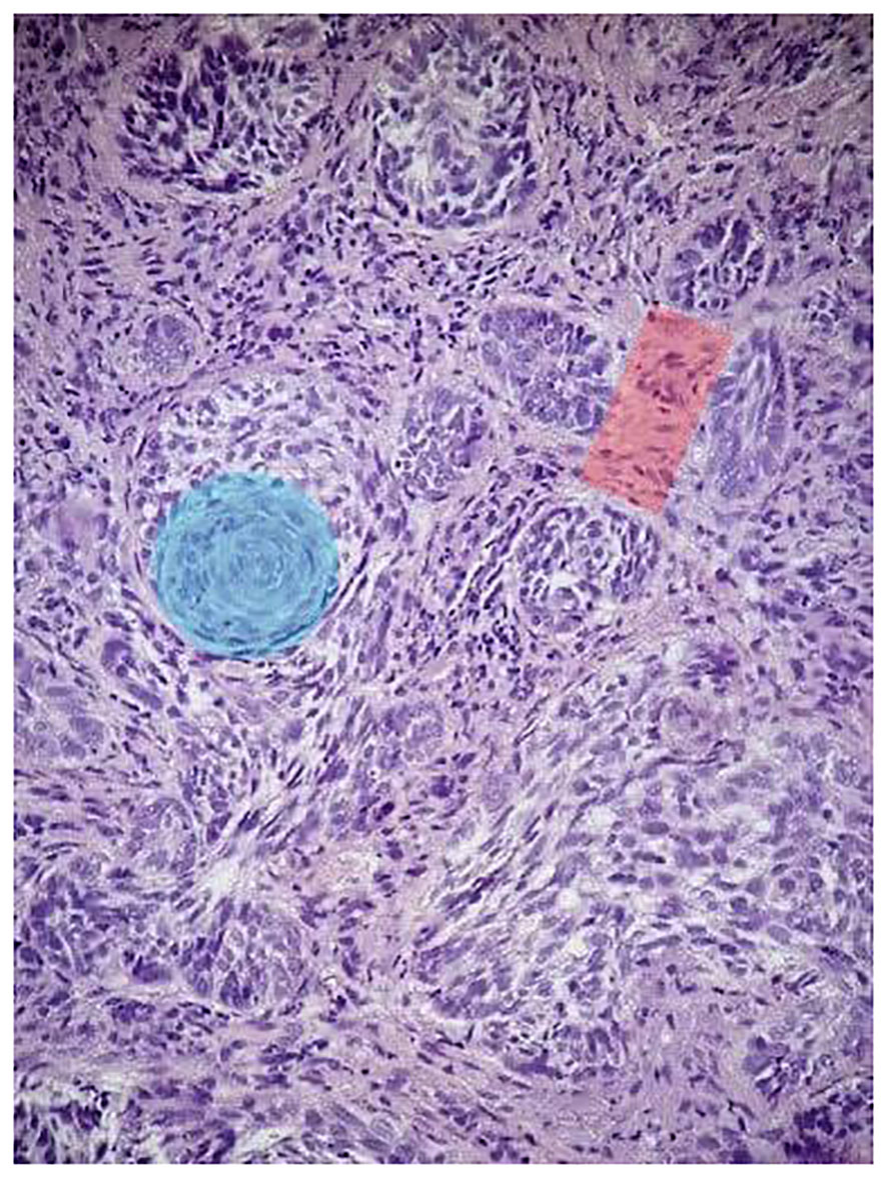
Histopathologic features of the patient’s nasal tip skin lesion punch biopsy. H&E (×10): the tumor is composed of a nest of basaloid cells with peripheral palisading (red shadow) and features of BCC admixed with areas of squamoid cells (blue shadow).

The patient noticed a nasal tip skin lesion with recurrent bleeding. On examination, the lesion was on the tip of the nose, measured 3 cm, and was black in color with ulceration. Due to the suspicious nature of the lesion, a punch biopsy was performed, which revealed basosquamous carcinoma (**Figure 1**). Subsequently, a complete resection of the lesion was performed with negative margins, followed by nasal reconstruction.

Six months later, the patient presented with lower urinary tract symptoms, including recurrent painless gross hematuria, urinary retention, frequency, weak stream, and nocturia. A physical examination was normal. Lab tests showed elevated alkaline phosphatase, gamma-glutamyl transferase, and alpha-fetoprotein (AFP) of 1,283.0 ng/ml and total prostate-specific antigen (PSA) of 70.6 ng/ml. Leukopenia and progressive thrombocytopenia were reported as well.

**Figure 2:**
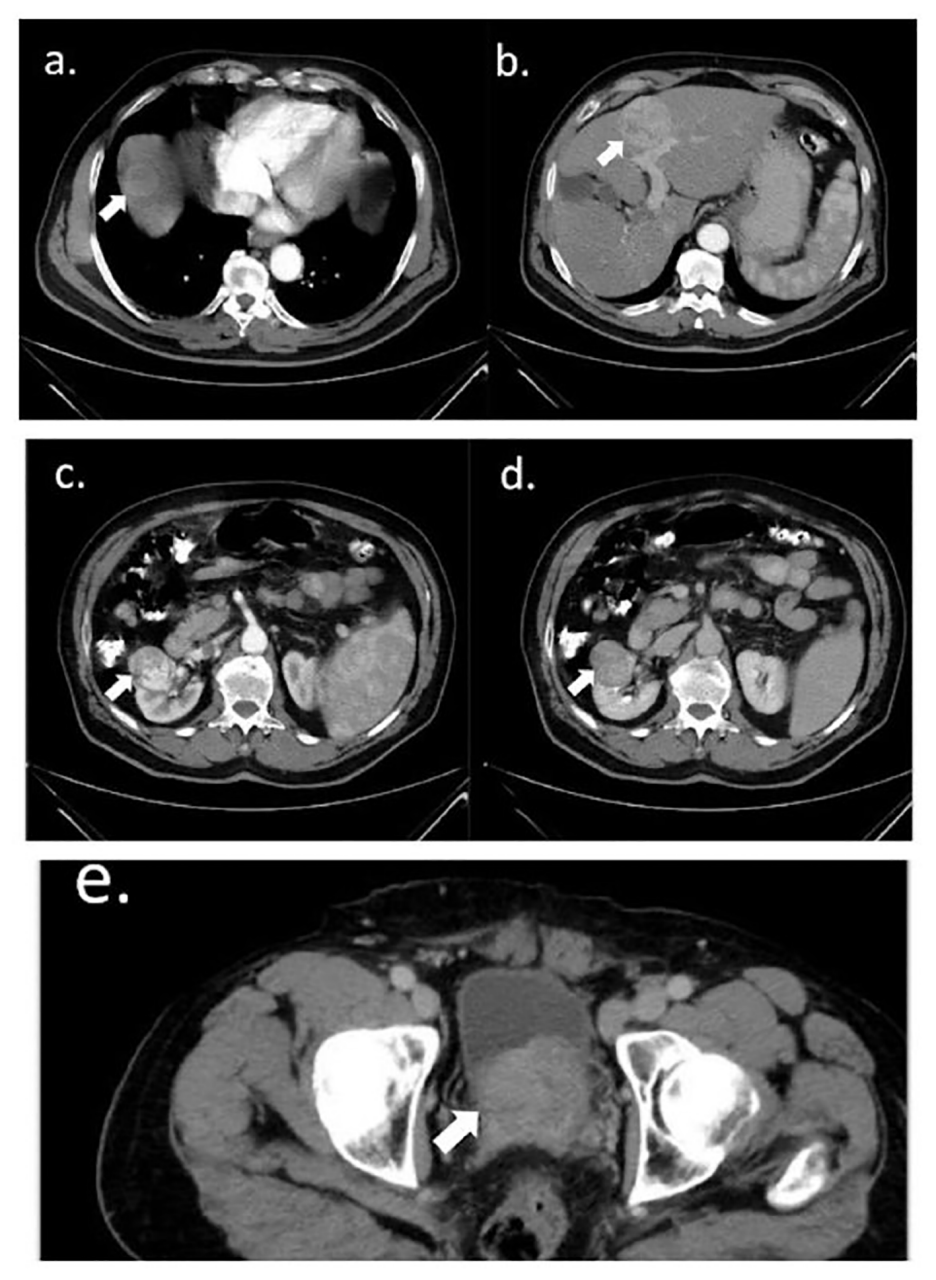
Abdomen and pelvis axial CT scan. **(A, B)** Hepatic isodense round lesions in the right lobe: a 5-cm lesion in segment four **(A)** and a 3.5-cm lesion in the dome of the liver **(B)**, with heterogeneous enhancement in the portal phase. In addition to two smaller enhanced lesions in segment eight **(B)**, the liver was normal in size and shape. **(C, D)** Right renal heterogeneously enhanced 4-cm lesion, arising from the right renal cortex, distorting adjacent mid-calyx and renal pelvis; **(C)** with and **(D)** without contrast. **(E)** An enlarged prostate of 6.3 cm projected to the base of the urinary bladder. All intended lesions are marked with arrows.

Chest, abdomen, and pelvis computed tomography (CT) scans revealed a markedly enlarged prostate gland indenting the urinary bladder; a right renal upper lobe solid mass arising from the cortex; and four enhanced hepatic lesions, mainly in the right lobe (**Figure 2**).

**Figure 3:**
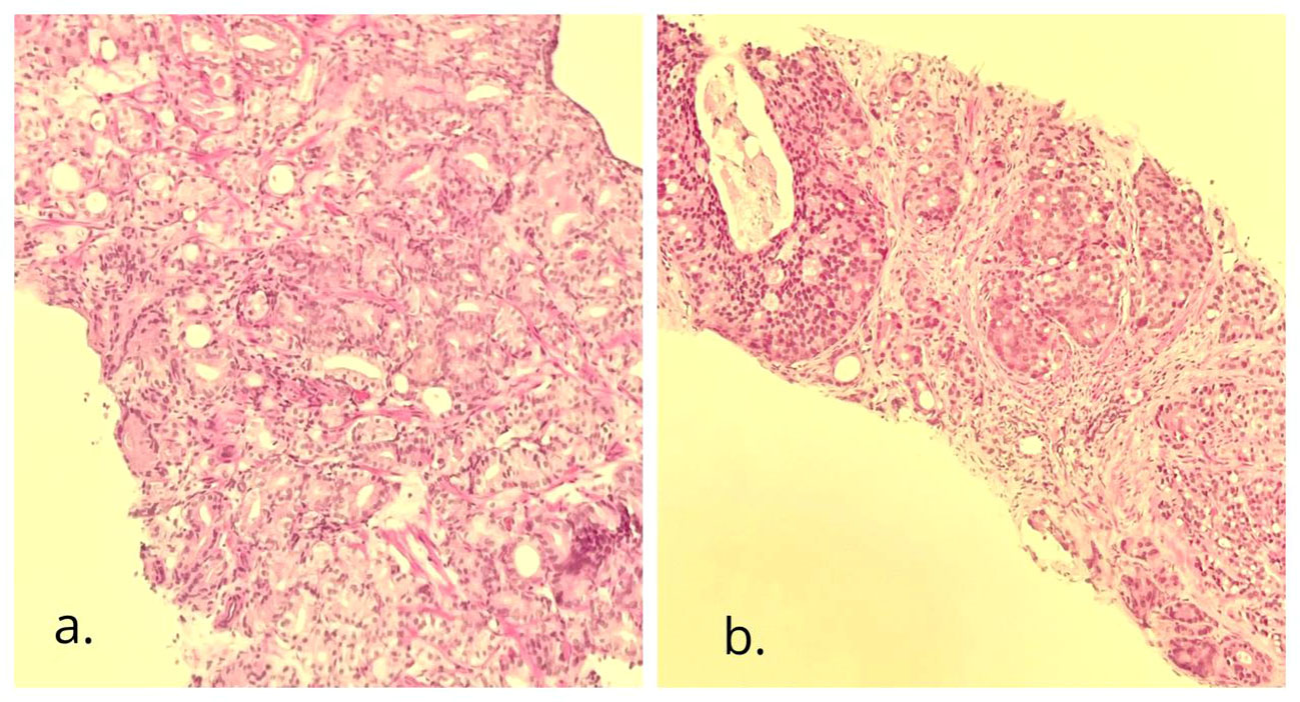
Needle biopsy of the prostate gland. H&E (×10): adenocarcinoma with cribriform pattern. **(A)** The right side of the gland with a Gleason score of 6 (3 + 3), grade group 1; **(B)** the left side of the gland with a Gleason score of 7 (4 + 3), grade group 3.

The urologists executed a transrectal prostate biopsy. Histopathology revealed adenocarcinoma, with a Gleason score of 6 (3 + 3) in the right parietal lobe and 7 (4 + 3) in the left parietal lobe (**Figure 3**).

**Figure 4:**
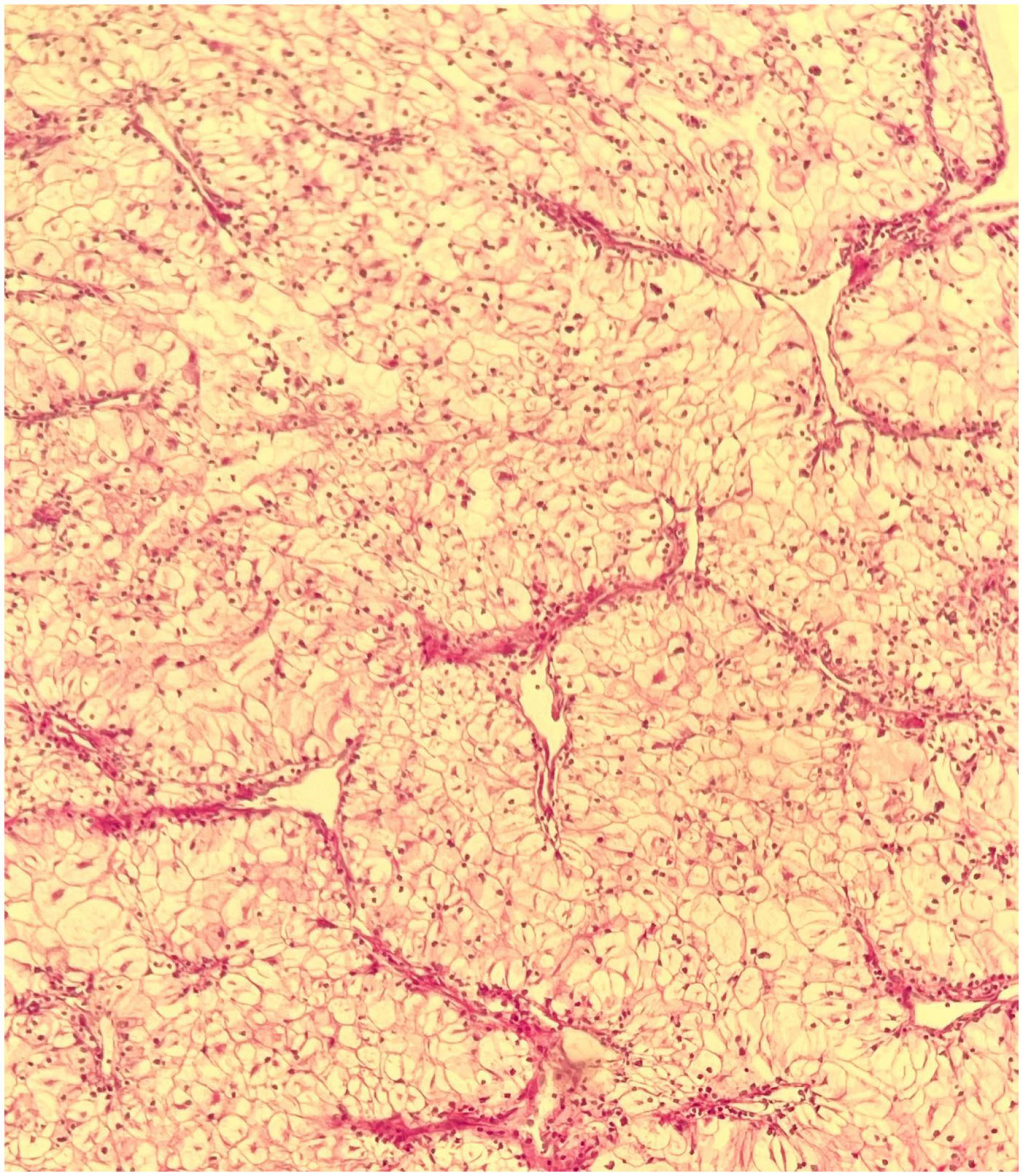
Biopsy of the right kidney tumor. H&E (×10): clear cell renal cell carcinoma, with WHO/ISUP histologic grade 3, and tumor pathologic stage of PT1b Nx Mx.

The renal mass was suspected to be renal cell carcinoma, leading the urologists to perform a right radical nephrectomy. Histologic examination of the tissue revealed a unifocal, 4.5 cm × 4 cm × 4 cm, WHO grade 3 clear cell renal carcinoma confined to the kidney (**Figure 4**).

**Figure 5:**
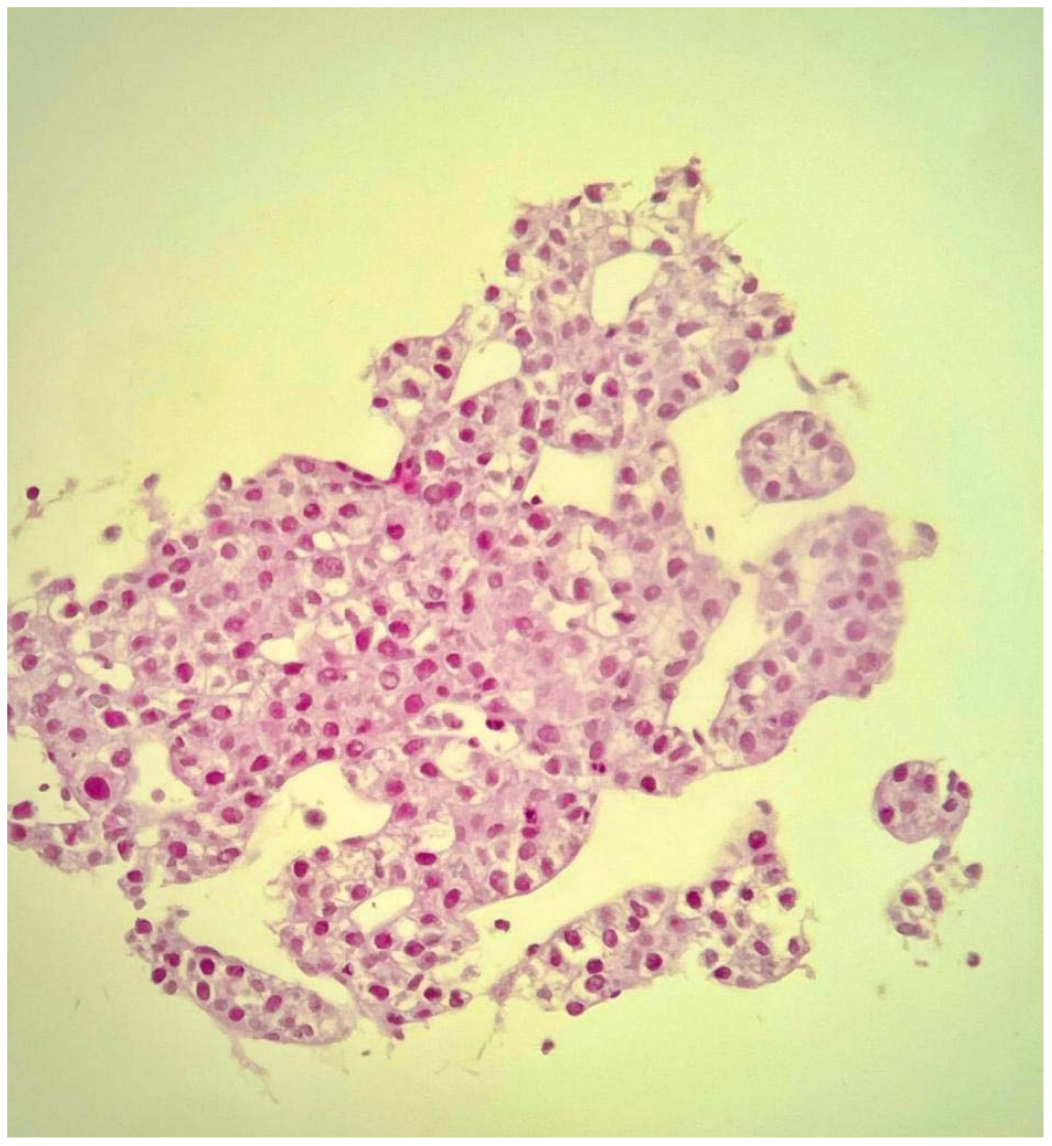
Core biopsy of the hepatic mass. H&E (×10): well-differentiated hepatocellular carcinoma.

An ultrasound-guided core needle biopsy of the largest hepatic lesion was also executed, and histopathology revealed well-differentiated hepatocellular carcinoma (**Figure 5**).

After discussing the patient’s findings, a multidisciplinary team decided to treat the prostate cancer with bicalutamide for 2 weeks, succeeded by goserelin, abiraterone acetate, and prednisolone. Hepatic lesions were infiltrative; hence, the decision was to treat the patient with atezolizumab and bevacizumab, starting 6 weeks after his radical nephrectomy. Regrettably, the patient failed to adhere to the prescribed management regimen for his hepatic malignancy. **Table 1** presents a comprehensive summary of the therapeutic modalities employed for our patient, encompassing indications, initiation time, dosages, and durations.

**Table 1:** Summary of the therapeutic modalities employed for our patient.

Treatment	Indication	Initiation time	Dosage	Duration
**Surgical resection with negative margins**	Nasal tip basosquamous cell carcinoma	1/2022	NA	NA
**Radical nephrectomy**	Clear renal cell carcinoma	7/2022	NA	NA
**Bicalutamide followed by goserelin, abiraterone, and prednisolone**	Prostatic adenocarcinoma	8/2022	**Bicalutamide:** 50 mg once daily for two weeks PO, followed by: **Goserelin:** 3.5 mg once monthly **Abiraterone:** 1000 mg daily, and **Prednisolone:** 5 mg twice daily	To be determined after re-evaluation.
**Atezolizumab and bevacizumab**	Hepatocellular carcinoma	The patient neglected his hepatic malignancy treatment	NA	NA

NA, Non Available.

One year later, a positron emission tomography (PET) scan exhibited a stable state of hepatic lesions; hypermetabolic malignant intraprostatic multifocal areas, invading the right seminal vesicles; hypermetabolic malignant left external iliac lymph nodes; hypermetabolic L1 spinous process lytic lesions; and nonmetabolically active multiple pelvic bone sclerotic lesions. The findings indicated possible prostate metastatic deposits; hence, a prostate-specific membrane antigen (PSMA) PET scan was planned to confirm the diagnosis. Genetic testing, BRCA gene testing, and Oncomine Pan-Cancer Cell-Free Assay reported no mutations.

## 3 Narrative review and discussion

### 3.1 Definition of MPMNs

Multiple primary malignancies are defined as the presence of more than one cancer in a single individual with exclusion to metastasis, recurrence, or local spread. According to the SEER project and the IACR/IARC, there are two distinct definitions (7). SEER categorizes tumors as synchronous if they develop within 2 months of the previous cancer diagnosis and as metachronous if they occur after that period (10). Additionally, SEER considers a single tumor in different parts of the same organ as multiple sites (5). In contrast, IARC rules are more exclusive; it uses a 6-month window to differentiate between synchronous and metachronous tumors (10). Furthermore, the IARC follows the one-site definition, where only one tumor is registered for a specific organ (2).

### 3.2 Incidence of multiple primaries

The literature shows that the overall incidence of MPMNs ranges widely from 2% to 17% (11), depending on the definition used, the analysis type, the data collection duration, the follow-up time, and the patient’s ethnicity (10). Warren first described the concept of multiple primary malignancies as early as 1932. Most studies express two cancers rather than triple or quadruple. Watanabe et al. noted second primaries in 5.2% of the cases and only 1.1% with triple or more cancers (12). Németh et al. reported the incidence of triple and quadruple primaries as 0.5% and 0.3% of cancer patients, respectively (13). In Antal and Vallent’s study, around 49 patients with MPMNs consisted of two cancers, and only four cases had three primary cancers. Furthermore, the majority of MPMNs were metachronous rather than synchronous (3). Therefore, our case is unique in that the patient presented with four synchronized primary malignancies, each with a different histopathology: nasal skin basosquamous carcinoma, which is considered a rare malignancy; prostate adenocarcinoma; hepatocellular carcinoma; and clear cell renal cell carcinoma. After thoroughly reviewing the literature, we concluded that this is the first case of this combination of malignancies. In addition, there was no proven association between them.

The incidence rates of MPMNs are on the rise. This is primarily attributed to the advancements in diagnostic methods’ sensitivities and the implementation of screening programs, especially for prostate and breast cancer (5, 6). Furthermore, the improved understanding of shared genetic and behavioral risk factors, among other factors, has contributed to this rise (3). This increase in incidence can also be attributed to the continuous evolution of treatment modalities, resulting in enhanced survival rates for cancer patients (6). An example of the progression in screening programs is the recent implementation of mammography, which is likely to result in an increase in the incidence of breast cancer (6). Screening programs have also pointed up new cases, particularly asymptomatic ones with low stages (10).

### 3.3 Etiology and risk factors

Risk factors of MPMNs involve host, lifestyle, environmental, and genetic factors: host factors include men (14), advanced age (5), black patients (14), and immune function; lifestyle include smoking, excessive alcohol use (14), obesity (15), physical inactivity, dietary patterns (16); environmental factors include previous cancer therapy, carcinogens, hormones, and infections; genetics factors represent family history, Caucasian ethnicity, and genetic mutations (10).

Patients over 50 years old account for more than 75% of MPMN cases due to prolonged carcinogenesis, immune attenuation, and increased cytokine production (17). Smokers are at increased risk for multiple primaries (10), especially stomach, liver, pancreas, kidney, uterine cervix, and myeloid leukemia (16), while excess alcohol use is related to cancers of the oral cavity, esophagus, colorectal, liver, and breast (16). Obesity is linked to an increased risk of endometrial and colon cancers (10). On the other hand, physical inactivity increases the risk of colon, breast, and endometrial cancers (16).

Prior cancer therapies, including chemotherapy and radiotherapy, contribute to multiple tissue injuries (18) and increase the risk of leukemia and lymphoma within the first 5 years and solid tumors after 5 years (19). Moreover, breast cancer hormonal therapy, like tamoxifen, increases the risk of endometrial cancer (20).

Immunodeficiency syndromes, either acquired or inherited, have a role in MPMNs (21). Human papillomavirus (HPV) infections represent the main cause of uterine cervical cancer as well as affect the anogenital tract, including the vulva, vagina, perineum, anus, and penis. In addition, oropharyngeal malignancies have been linked to HPV-16 (22). Patients with the human immunodeficiency virus (HIV) are more likely to develop cervical and anal cancer, non-Hodgkin lymphoma, and Kaposi sarcoma. In Palestine, the prevalence of HPV and HIV is considered low, so routine screening for these infections is not typically conducted.

Multiple primary tumors sometimes present as familial cancer syndromes that account for 1% to 2% of all cancers. They include multiple endocrine neoplasia types 1 and 2, von Hippel–Lindau disease, hereditary breast and ovarian cancer syndrome, and others (5). The role of genetics in the development of cancer has revealed more than 100 mutant predisposing genes (23). Patients with familial cancer syndromes face a 3% risk of developing a second primary cancer each year after their initial cancer diagnosis (24).

Regarding childhood cancers, more than a quarter of survivors suffer from new cancers (25). Furthermore, the incidence of cancer patients who are between 60 and 69 years old developing a second cancer is 13% (11). Studies have shown that renal cell carcinoma and lymphoma are recently occurring more frequently as second primaries compared to their occurrence as first primaries (26).

Another important risk factor is ethnicity. For example, Japan has a high incidence of stomach cancer but a low incidence of prostate cancer (27). Additionally, patients of Caucasian origin have higher frequencies of multiple primaries in contrast to other ethnicities (10).

In the present case, the patient’s age and gender may be potential risk factors. His family history is significant for breast and endometrial cancer in his sister, but there is no evidence suggestive of a hereditary cancer syndrome; he also has no history of chemotherapy or radiation exposure; and he does not smoke or drink alcohol. Regarding genetic testing results, BRCA gene testing and Oncomine Pan-Cancer Cell-Free Assay results were negative. Oncomine Focus Assay, however, targets a panel of 52 genes. A comprehensive genetic sequencing could inspect other mutations that are not targeted by the oncomine assay.

### 3.4 Diagnosis

Knowing that malignancy survivors are at increased risk for subsequent tumors (8, 10), a comprehensive clinical assessment, including sophisticated imaging studies such as PET-CT scan and whole-body MRIs, should be executed for proper tumor staging and detection of other possible primaries. Furthermore, patients should be followed up regularly using guideline-based plans and recommended screening programs (2, 14, 23).

Alexia et al. have listed several clinical features indicating the possibility of a second primary tumor: atypical metastatic pattern of a primary tumor; disproportionate tumor burden and tumor marker load; markedly late onset for metastasis; and a single new metastatic lesion, among other features (14).

Pathological confirmation and independent staging and evaluation for each tumor should be pursued if MPMN patients are considered for active treatment. The primary tissue should always be available for comparison, especially in cases of undifferentiated tumor histology. In addition, clinicians should collect more than one specimen from a patient with multiple metastatic sites (2, 14).

When clinicians suspect a hereditary cancer phenomenon (e.g., several family members with MPMNs or certain cancers affecting young family members for several generations) (5), genetic testing can be informative. Currently, gene panels can assess most tumor-predisposing mutated genes (23), which can guide patient management (i.e., novel targeted agents and checkpoint inhibitors) (14), in addition to a follow-up plan and the need for further testing for patients’ relatives (14, 23). Cezary et al. discussed the indications, advantages, and limitations of genetic testing for MPMN patients (23).

### 3.5 Treatment

Therapeutic options for patients with multiple primaries are rarely discussed, besides the exclusion of these cases from most clinical trials involving novel treatments. Furthermore, available drug–drug interaction data for cytotoxic, biologic, and immunotherapy cancer treatments are neither reliable nor efficient.

To ensure an appropriate treatment plan, a MDT should discuss an individualized therapeutic strategy for each patient depending on the pathological type, the stage of each tumor, and the patient’s physical condition, besides plan adaptation as needed. The patient should be aware of treatment challenges, predicted prognosis, and possible signs and symptoms for recurrent or second primary tumors (5, 14). It is recommended to treat MPMN patients with an aggressive strategy due to potential long-term survival, except for elderly and asymptomatic patients with more than three primaries (14).

For localized tumors, surgery, radiation, or chemoradiation may be suitable for two primaries. However, for more advanced tumors, or in the case of more than two primaries, the challenge is to find a therapeutic strategy, mostly systemic therapies, that covers all cancer types without increasing toxicity, possible pharmacological interactions, or worsening the prognosis. Alexia et al. have discussed points to take into consideration while treating synchronous versus metachronous multiple primaries (14).

As a general rule, the tumor with the greatest contribution to the patient’s survival or quality of life should be prioritized in the treatment. Surgical resection, accompanied by adjuvant therapy for fitting tumors, should be a priority. In cases where single chemotherapy is not appropriate for all MPMNs, the chemotherapy controlling later-stage malignancy is prioritized (2).

Regarding the treatment plan implemented for our patient, his nasal tip basosquamous cell carcinoma was managed by surgical resection with negative margins, obviating the necessity for adjunctive therapy. Six months later, the patient underwent a right radical nephrectomy for an incidentally discovered renal mass in the right renal cortex, with histopathology of clear cell renal cell carcinoma. Given the tumor’s staging, adjuvant therapy was deemed unnecessary. For the synchronous prostate adenocarcinoma of stage IV with bone metastasis, following nephrectomy, he was initiated on a therapeutic regimen consisting of bicalutamide hormone therapy for 2 weeks, followed by goserelin and abiraterone hormone therapies with prednisolone. In consideration of his synchronous hepatocellular carcinoma, the discovered masses were infiltrative and unresectable; therefore, MDT decided to treat the patient with atezolizumab immunotherapy and bevacizumab antiangiogenic drug, scheduled 6 weeks postnephrectomy, but the patient lost follow-up for his hepatic malignancy treatment plan. At the 1-year follow-up assessment, there was no evidence of recurrence of the nasal skin lesion or renal carcinoma. PET imaging also reported stable hepatic masses and evidence of metastatic lesions involving the L1 spinous process and pelvic bones. Remarkably, the PSA levels demonstrated a substantial reduction from 70.6 to 8 ng/ml.3.6.

### 3.6 Prognosis

Survival of patients with multiple primary neoplasms varies and is influenced by cancer origin, tumor stage, onset, and site of consecutive primaries (10) (i.e., hepatocellular cancer being a synchronous primary tumor carries the worst prognosis (25)). Other factors influencing the outcome include genetics, the patient’s lifestyle, and comorbidities (10).

Amer has stated that patients with multiple primaries have a much higher survival rate compared with single tumor patients, with the life expectancy for patients with three or more primaries being similar to that of age- and sex-matched normal population (10). Moreover, the longer the time between the first and second primaries, the better the outcome. Correspondingly, metachronous primaries have a better prognosis in comparison with synchronous primaries (10). On the other hand, Min Yi et al. have concluded from their study on breast cancer patients with MPMNs that MPMNs have a worse prognosis compared with a single primary tumor (15).

In addition to proper investigations and follow-up of the primary tumor and potential treatment adverse effects, cancer survivors should be guided with cancer prevention and early detection recommendations, including smoking cessation, a healthy diet, and physical activity (5).

## 4 Conclusion

Multiple primary malignant neoplasms are no longer a rare entity, yet the topic lacks proper explanations and guidelines. There is wide variability regarding incidence and different methods in describing definitions. Furthermore, the data in the literature has discussed possible varying theories considering etiologies and significant risk factors. Eventually, diagnostic approaches consistent with single primaries in MPMN patients may lead to a delay in the detection of further malignancies and miss possible underlying genetic predisposition.

Therefore, a standardized reporting protocol is needed to detect the precise representing definition and incidence. In addition, patients with MPMNs require cancer screening programs since they are a high-risk population. Moreover, guidelines for treatment techniques that include therapeutic prioritizations, possible drug interactions, long-term adverse effects, and predicted outcomes are necessary. Lastly, reports in the literature addressing individualized cases and their workup approaches with reported outcomes could be carefully applied to similar situations with the intention of providing practitioners with valid guidelines.

## Data availability statement

The original contributions presented in the study are included in the article/Supplementary Material. Further inquiries can be directed to the corresponding author.

## Ethics statement

Written informed consent was obtained from the individual(s) for the publication of any potentially identifiable images or data included in this article.

## Author contributions

RSa: Writing – original draft, Writing – review & editing. ZG: Writing – original draft, Writing – review & editing. WB: Project administration, Supervision, Writing – review & editing. RS-A: Project administration, Supervision, Writing – review & editing. HH: Project administration, Supervision, Writing – review & editing. RSw: Supervision, Writing – review & editing. IB: Supervision, Writing – review & editing.
